# Supplementary material for: A second photoactivatable state of the anion-conducting channelrhodopsin GtACR1 empowers persistent activity
Source: Commun Biol. 2025 Aug 8;8:1183. doi: 10.1038/s42003-025-08560-4 (PMC12334634; doi:10.1038/s42003-025-08560-4)
Supplement: Supplementary file 1 — Supplementary Information [file 42003_2025_8560_MOESM1_ESM.pdf]

Supplementary Information

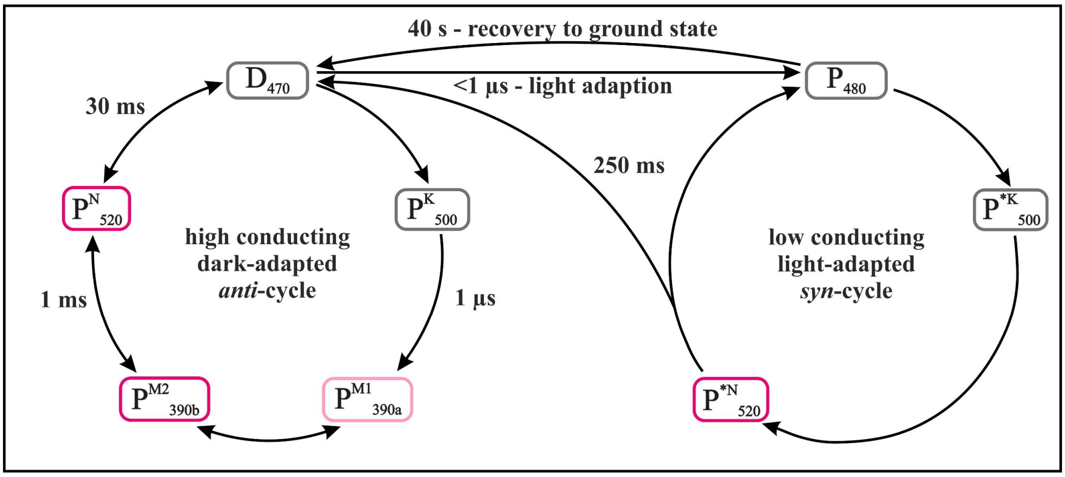

Supplementary Figure 1: Photocycle model of *Cr*ChR2 Model of the branched photocycle of *Cr*ChR2 (reproduced and modified from Kuhne et al.[1]). The "dark-adapted" *anti*-cycle (left) exclusively comprises a C=N-*anti*-configuration of the retinal, is highly conducting and relatively fast. In the "light-adapted" *syn*-cycle (right), the retinal has a 13-*cis*, C=N-*syn* configuration. The *syn*-cycle is poorly conducting and decays at a slower rate. During continuous illumination, molecules are accumulated in the *syn*-cycle, which causes the inactivation of *Cr*ChR2.

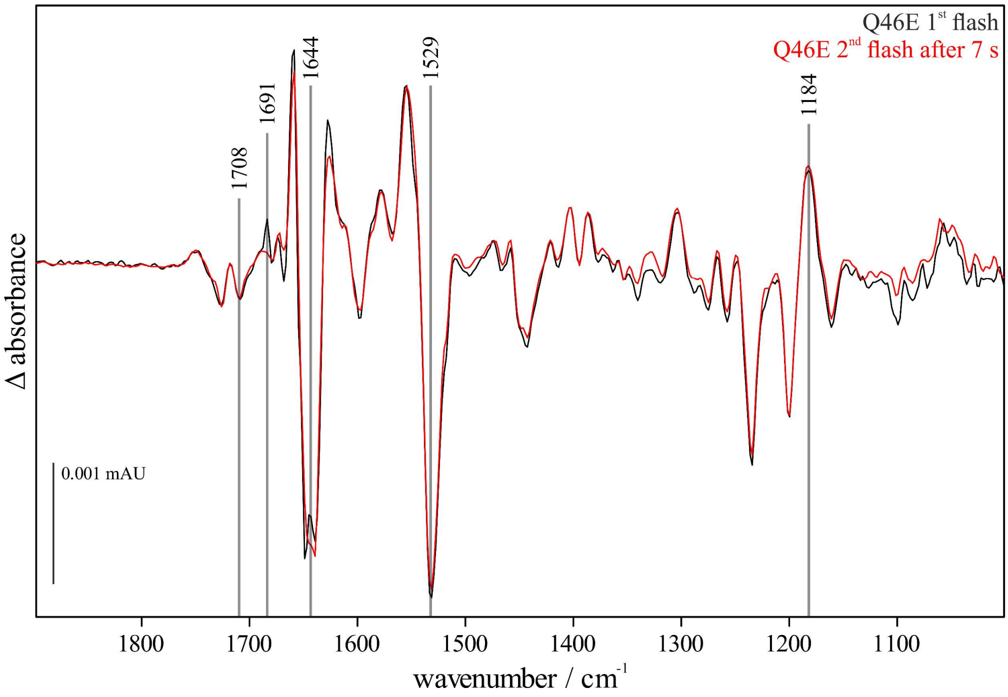

Supplementary Figure 2: Excitation spectra of the *Gt*ACR1 variant Q46E at different exposure times. The respective excitation spectra after one flash (black) and a second flash after 7 s (red) are shown. Similar to the *Gt*ACR1 WT, a large proportion of the proteins can be re-excited after 7 s. The time of the second flash was adapted to the lifetime of the mutant (see Supplementary Note 4 and Supplementary Table 1).

Supplementary Table 1: Determined half-lives $t_{1/2}$ of all analysed samples obtained by rapid-scan FTIR measurements

**Sample name**	**T_4/ ms**	**T_5/ ms**	**T_6/ ms**
ACR1 WT 1st flash	44	150	15890
ACR1 WT 2nd flash	88	1438	93490
ACR1 WT 5th flash	76	1849	68960
ACR1 WT continuous light	203	3415	68290
ACR1 Q46E 1st flash	230	965	30570
ACR1 Q46E 2nd flash	171	2285	667100

Supplementary Table 2: FTIR marker bands that are important in connection with retinal proteins

**Wavenumber / cm^{-1}**	**associated group**
1184	protonated 13-*cis* retinal
1691	pore formation, channel opening and conducting state
1708	protonated E68
1644	conformational changes during channel opening and closing
1529	retinal C=C ground state
1444	M intermediate

Supplementary Note 1:

In order to be able to make reliable statements about the excitability of the O intermediate, it was necessary to determine how much of the sample is excited upon one flash.

With help of the background spectrum, which reflects the entirety of the proteins in the sample in the amide II band, and the excitation spectrum, which shows the excited proteins, the percentage excitation could be calculated using the Lambert-Beer law as follows:

In a first step a solution containing 100 mM all-*trans* retinal was measured on a micro ATR and the molar extinction coefficient of retinal was calculated approximately using the Lambert-Beer law

$$\varepsilon_{Ret} = \frac{A_{1375\,cm^{-1}}}{c \times d} = \frac{0.213}{0.1\,M \times 0.0009527\,cm} = 2200\,\frac{1}{M \times cm}$$

A similar value is found in the literature[2].

Whereby the effective penetration depth (d_e) of the micro-ATR cell was calculated with the penetration depth of one reflection (d_p) and the number of total reflections (N):

$$d_p = \frac{\lambda}{2\pi n_1 \sqrt{sin^2\theta - (\frac{n_2}{n_1})^2}} = \frac{7256\,nm}{2\pi \times 2.4\sqrt{sin^2(45°) - (\frac{1.3}{2.4})^2}} = 1058\,nm$$

$$d_e = N \times d_p = 9 \times 1058\,nm = 9527\,nm$$

The extinction coefficient of the amide II band (ε_{AII}) is calculated from the number of peptide bonds (541) of the protein and the extinction coefficient of a single molecular vibration

$$\varepsilon_{AII} = 350\,\frac{1}{M \times cm} \times 541 = 189350\,\frac{1}{M \times cm}$$

The amide II absorbance of the total protein was determined with

$$A = -lg\frac{I}{I_0} = -lg\frac{0.32}{0.66} = 0.31$$

The absorbance change of the excited protein could be read in the excitation spectrum at 1235 cm^{-1}:

$$\Delta A = 0.0017$$

Finally, the ratio of excited protein to total protein can be calculated:

$$ratio\ excitation = \frac{c_{excited} \times d}{c_{total} \times d} = \frac{\Delta A}{A} \times \frac{\varepsilon_{A2}}{\varepsilon_{Ret}} = \frac{0.0017}{0.31} \times \frac{189350}{2200} = 47\ \%$$

In this way, the excitation of 6 samples was calculated, whereby an average excitation of the sample upon one flash of about 41 % +- 5 % was determined.

Subsequently, before the 5th flash $0.6^4 = 13$ % of the sample remain in the ground state, which means that 87 % are excited from the O intermediate.

Supplementary Note 2:

The time at which 99% of the M intermediate was decayed was calculated as follows.

First, the decay constant k was calculated using the half-life ($t_{1/2}$) for the decay of M (T_5) as determined by the FTIR measurements

$$k = \frac{\ln(2)}{t_{\frac{1}{2}}} = \frac{\ln(2)}{150\,ms} = 0{,}0046\,ms^{-1}$$

Finally, the point in time was calculated at which only 1% of the intermediate ($A = \frac{1}{100}A_0$) was still present

$$A = A_0 \cdot e^{-kt} \leftrightarrow t = \frac{\ln\left(\frac{A}{A_0}\right)}{-k} = \frac{\ln\left(\frac{1}{100}\right)}{-\,0{,}0046\,ms^{-1}} = 996\,ms$$

According to this calculation, 99% of the M intermediate should have decayed after 996 ms, i.e. 0.99 s.

Supplementary Note 3:

The percentage of the ground state and resulting from this the percentage of the N/O intermediate of the protein that enters the photocycle was calculated as follows.

First, the decay constant k was calculated using the half-life ($t_{1/2}$) for the decay of N/O (T_6) as determined by the FTIR measurements

$$k = \frac{\ln(2)}{t_{\frac{1}{2}}} = \frac{\ln(2)}{15890\,ms} = 4.36 \times 10^{-5}\,ms$$

Finally, the percentage of how much N/O intermediate is still present after 1 s was calculated

$$A = A_0 \cdot e^{-kt} \leftrightarrow \frac{A}{A_0} =\cdot\, e^{-k} = e^{4.36\times10^{-5}\,ms^{-1}\times1000\,ms} = 0.96$$

According to this calculation, 96% of the proteins is still in N/O intermediate 1 s after the initial excitation, correspondingly 4% have already reached the ground state.

Supplementary Note 4:

The time at which 99% of the M intermediate in the *Gt*ACR1 variant Q46E was decayed was calculated as follows.

First, the decay constant k was calculated using the half-life ($t_{1/2}$) for the decay of M (T_5) as determined by the FTIR measurements

$$k = \frac{\ln(2)}{t_{\frac{1}{2}}} = \frac{\ln(2)}{965\ ms} = 0{,}0007\ ms^{-1}$$

Finally, the point in time was calculated at which only 1% of the intermediate ($A = \frac{1}{100} A_0$) was still present

$$A = A_0 \cdot e^{-kt} \leftrightarrow t = \frac{\ln\left(\frac{A}{A_0}\right)}{-k} = \frac{\ln\left(\frac{1}{100}\right)}{-\ 0{,}0007\ ms^{-1}} = 6579\ ms$$

According to this calculation, 99% of the M intermediate should have decayed after 6579 ms, i.e. 6.6 s.

References

1 Kuhne J, Vierock J, Tennigkeit SA, et al. Unifying photocycle model for light adaptation and temporal evolution of cation conductance in channelrhodopsin-2. *Proc Natl Acad Sci U S A*. 2019;116(19):9380-9389. doi:10.1073/pnas.1818707116.

2 Andresen ER, Hamm P. Site-specific difference 2D-IR spectroscopy of bacteriorhodopsin. *J Phys Chem B*. 2009;113(18):6520-6527. doi:10.1021/jp810397u.
