# Supplementary material for: A second photoactivatable state of the anion-conducting channelrhodopsin GtACR1 empowers persistent activity
Source: Commun Biol. 2025 Aug 8;8:1183. doi: 10.1038/s42003-025-08560-4 (PMC12334634; doi:10.1038/s42003-025-08560-4)
Supplement: Supplementary file 2 — Description of Additional Supplementary Files [file 42003_2025_8560_MOESM2_ESM.pdf]

Description of Additional Supplementary Files

File name- Supplementary data 1

File description- The source data behind the graphs in the paper
